# Intravesical Injection of Abobotulinumtoxin-A in Patients with Bladder Pain Syndrome/Interstitial Cystitis

**DOI:** 10.5152/tud.2023.22243

**Published:** 2023-05-01

**Authors:** Mohammad-Sajjad Rahnama’i, Hanieh Salehi-Pourmehr, Sana Saeedi, Sona Tayebi, Sakineh Hajebrahimi

**Affiliations:** 1 Department of Urology, St. Elisabeth-Tweesteden Hospital, Tilburg, the Netherlands; 2 Research center for Evidence-Based Medicine, Iranian EBM Center: A Joanna Briggs Institute Center of Excellence, Tabriz University of Medical Sciences, Tabriz, Iran; 3 Student Research Committee, Tabriz University of Medical Sciences, Tabriz, Iran; 4 Department of Urology, Ahvaz Jundishapur University of Medical Sciences, Ahwaz, Iran; 5 Department of Urology, Faculty of Medicine, Tabriz University of Medical Sciences, Tabriz, Iran

**Keywords:** Abobotulinumtoxin-A, cystitis, interstitial, bladder pain syndrome, botulinum toxins, type A

## Abstract

**Objective:**

This study aimed to evaluate retrospectively the outcomes of Abobotulinumtoxin-A (Dysport®) intravesical injection in refractory interstitial cystitis/bladder pain syndrome patients to first- and second-line treatment.

**Materials and Methods:**

From March 2016 to 2021, 44 adult patients with bladder pain syndrome who were refractory to first- and second-line treatment were enrolled in our study. The Bladder Pain/Interstitial Cystitis Symptom Score questionnaire was filled out for every patient before and 1-3 months after intervention in addition to urodynamic evaluation. Patient satisfaction was evaluated using a scoring system that was defined as high or >80% improvement (highly satisfied), intermediate 40%-79% (intermediate satisfaction), and poor 0%-39% improvement.

**Results:**

The mean age of our study population was 57 years, including 41 females and 3 males. The mean follow-up time was 9 months. According to the results of urodynamics, 68% of cases had low capacity, and detrusor overactivity, while 18% had only low capacity. In terms of the endpoint outcome, half of the patients (52%) had intermediate satisfaction, whereas 41% reported a good response. Only 3 cases had no response or felt (7%) any improvement after the intervention (poor response). The paired *t*-test analysis revealed that the mean Bladder Pain/Interstitial Cystitis Symptom Score was reduced after injection (*P* = .001).

**Conclusion:**

Our results showed the efficacy and safety of intravesical injections with Abobotulinumtoxin-A (Dysport®) in patients with interstitial cystitis/bladder pain syndrome. Further randomized controlled trials are recommended to investigate its superiority over placebo considering the need for anesthesia, the occurrence of local complications, risks of urinary retention, and a large post-void residual (PVR) volume.

**Main Points**

- Recent studies have shown that botulinum toxin A injections can reduce inflammation and thus improve the protective function of urothelium in patients with interstitial cystitis/bladder pain syndrome (IC/BPS).
- Our results showed that intravesical injections with Abobotulinumtoxin-A (Dysport®) in patients with IC/BPS were effective and safe.
- This therapeutic method has a high rate of satisfaction (41% highly satisfied and 52% intermediate satisfaction).
- Further randomized controlled trials are recommended to investigate Dysport® superiority over placebo considering the need for anesthesia, the occurrence of local complications, risks of urinary retention, and a large post-void residual volume.

## Introduction

About 6.5% of the 8 million women over the age of 18 in the United States are estimated to have interstitial cystitis/bladder pain syndrome (IC/BPS).^1^ Interstitial cystitis/bladder pain syndrome, recurrent or persistent chronic pelvic pain, is a feeling of pressure or discomfort associated with the bladder and has at least one other urinary tract symptom, such as an urgent need to urinate or frequent urination.^2^ The exact pathogenesis of painful bladder syndrome is not yet clear. Common histopathological findings include denuded urothelium mucosal ulcers, submucosal inflammation, granulation, and neuron-mediated inflammation that might trigger secretion from mast cells, indicating an inflammatory process.^3^ Increased apoptosis and abnormal E-cadherin expression in the urothelium of urothelial cells are the results of suburothelial inflammation,^4,5^ which was reported in the last years. When the bladder is stretched, ATP released from the urothelium can activate ion channels containing the P2X3 receptor, which stimulates neuronal recharging.^6^ Inflammation may affect the supply of neuropeptides and increase bladder irritation and sensitivity, which leads to increased pain sensation during bladder distance.^7^

Medical history, the used medication (including ketamine), and surgery history should be considered to rule out any other cause of pelvic pain. The general principles for treating patients are to improve their quality of life and meet their real demands. Behavior modification, including scheduled urination, adequate fluid intake, and bladder exercise, has been suggested as the first line of treatment. Pharmacotherapy has been approved as the second line of treatment. Amitriptyline is recommended in most guidelines. Intravesical treatment methods including intravesical implants of anti-inflammatory drugs, analgesics, and substances that replace the defective glycosaminoglycan layer of the bladder have been studied. Cystoscopic hydrodistension of the bladder and the destruction of Hunner lesions is the third line of treatment. OnabotulinumtoxinA (BoNT-A) and sacral neuromodulation are the other promising therapeutic approaches in IC/BPS.^8^ The gram-positive, anaerobic bacteria *Clostridium botulinum* produces the strong neurotoxin known as botulinum toxin (BoNT). Types A, B, C1, D, E, F, and G are the 7 known immunologically different serotypes of BoNT. The 150 kDa polypeptide chain that makes up Clostridium neurotoxins is cleaved into 2 active molecules by tissue proteinases: a heavy chain (H) of about 100 kDa and a light chain (L) of about 50 kDa kept together by a single disulfide bond. Each serotype has a unique range of mechanisms of action and time course of effect. Botulinum toxin serotype’s heavy chain attaches to a particular neuronal ecto-acceptor, which causes membrane translocation and endocytosis by intracellular synaptic vesicles. As SNAP-25 is cleaved by the light chain, synaptic exocytosis is prevented, which prevents neuronal transmission. Most scientists agree that this function alone does not appear sufficient to explain the full of neurotoxin’s apparent analgesic impact. However, the action of BoNT to inhibit the release of acetylcholine at the neuromuscular junction is best recognized. Because of this, research and clinical evidence have shown that BoNT may have different antinociceptive pathways in a range of painful conditions.^9^ Recent studies have shown that botulinum toxin type A (BoNT-A) injections can reduce inflammation and thus improve the protective function of urothelium in patients.^10^ Possible mechanisms of BoNT-A acting in the treatment of BPS/IC include inhibition of detrusor muscle activity, sensory modulation, antinociceptive, and anti-inflammatory effects in the urothelium.^11^ Although previous studies have shown promising efficacy of BoNT-A single-dose injection in the treatment of BPS,^12,13^ the long-term effects have not been successful,^14^ and it is stated that the therapeutic duration was found to be longer with repeated BoNT-A injections than with a single injection.^15^ Botulinum toxin type A is an accepted therapy for several urologic diseases involving the lower urinary tract system. Besides BoNT-A-A, Abobotulinumtoxin-A (Dysport®) has been used to treat lower urinary tract symptoms (LUTS) as an off-label treatment. Since 2004, onabotulinumtoxinA has been used for treating IC/BPS. However, the only serotype available in our country is Dysport®. This study aimed to evaluate retrospectively the outcomes of Dysport® intravesical injection in refractory BPS patients to first and second-line treatment.

## Materials and Methods

This study has been carried out in compliance with all the rules and instructions related to medical research in Iran. Written consent was obtained from all participants in this project. This study was conducted after approval by the regional ethic committee of Tabriz University of Medical Sciences with the ethical code of (.1400.848).

From March 2016 to 2021, 44 adult patients with BPS who were refractory to first- and second-line treatment were enrolled in our study. The patients did not receive previous doses of Dysport®. The patients who had detrusor underactivity of bladder outlet obstruction (BOO) were excluded from the study.

The Bladder Pain/Interstitial Cystitis Symptom Score (BPIC-SS) questionnaire was filled out for every patient before and 1-3 months after intervention in addition to urodynamic evaluation. The BPIC-SS, which was developed and validated for a patient-reported symptom-based instrument, and used for clinical trial eligibility of BPS patients, had strong sensitivity (0.72) and specificity (0.86) with a cut score ≥ 19 to determine clinical trial inclusion.^16^ All patients were treated with an intravesical injection of 500 U of Abobotulinumtoxin-A (Dysport®).

In terms of bladder compliance, the definition was as follows: The difference between the change in detrusor pressure (*P*_det_) and the change in bladder volume (*V*) during filling cystometry is bladder compliance (*C*). *C* = Δ*V*/Δ*P*_det_.^17^ In non-neurogenic bladders, the normal compliance is > 40 mL/cm H_2_O, while low compliance is defined as < 30 mL/cm H_2_O; in neurogenic bladders, normal compliance is > 30 mL/cm H_2_O, and <10 mL/cm H_2_O is considered as low compliance.^18^

Although fairly varied, the typical bladder capacity is more than 300 mL. Therefore, if the bladder capacity is lower, irritable voiding symptoms will develop. In the current study, if the functional bladder capacity was less than 200 mL, it was defined as low bladder capacity.

Patient satisfaction was evaluated using a scoring system that was defined as high or >80% improvement (highly satisfied), intermediate 40%-79% (intermediate satisfaction), and poor 0%-39% improvement. Parametric and non-parametric statistical analysis was applied with a significant rate of the *P* value of < .05 using Statistical Package for Social Sciences statistical software version 24 (IBM SPSS Corp., Chicago, Ill, USA).

The ending outcome was categorized as the improvement of symptoms, good response to treatment, or no response to therapeutic approach.

## Results

The mean age of our study population was 57 years, including 41 females and 3 males.

The mean duration time of symptoms was 27 months (minimum of 6 months, and maximum of 120 months). Most of the cases were females (93%). The mean follow-up time was 9 months (range 3-48 months).

One dose of previous Abobotulinumtoxin-A injections was received by 16 cases, 3 patients received 2 doses, and 1 case was a candidate for 3 times injections.

The minimum interval between the previous Dysport® injection and the last one was at least 6 months. In addition, previous pharmacotherapy for eligible cases was oral medications (n = 31), oral pharmacotherapy, and percutaneous nerve evaluation (PNE) in 1 patient, and oral pharmacotherapy and intravesical injection in 12 patients.

The results of urodynamic studies in the eligible cases are summarized in Table 1. According to the results, 68% of cases had low capacity, and detrusor overactivity, while 18% had only low capacity.

In terms of the endpoint outcome, half of the patients (52%) had improvement following the treatment (intermediate satisfaction), whereas 41% reported a good response following treatment (highly satisfied). Only 3 cases had no response or felt (7%) any improvement after the intervention (poor response).

The paired *t*-test analysis revealed that the mean BPIC-SS score was 22.4, which was reduced to 6 after Abobotulinumtoxin-A injection (*P* = .001). Side effects were seen in 4 patients. One patient had persistent urinary tract infections, and 3 developed a voiding dysfunction for which clean intermittent self-catheterization (CIC) was advised. We analyzed the items of the BPIC-SS questionnaire, and according to the results, all items improved after treatment; however, we did not find any correlation between the pre-treatment score and end outcomes of patients (*P* >.05) according to the results of Spearman's rank-order correlation coefficient (Tables 2 and 3).

## Discussion

Our data show that the treatment is safe and has a high rate of satisfaction, that is, 41% highly satisfied and 52% intermediate satisfaction.

Initially, BoNT-A was the only botulinum toxin that was FDA-approved for use in patients with neurogenic detrusor overactivity (NDO).^19,20^ In 2009, Dysport® was also approved by the FDA for use in patients with overactive bladder (OAB).^21,22^ Dysport®, like BoNT-A, acts on nerve impulses, although its formula is slightly different and contains smaller components. The safety, effectiveness, and quality of both products are the same.^23^ Both BoNT-A and Dysport® toxins are type A serotypes, despite differences in the species of bacteria that produce them, isolation of the manufacturing process, purification, and extraction.^24^ This makes a difference in the doses required for these 2 types of toxins (converting BOTOX® to Dysport® at a ratio of 2 : 3 : 1, although this ratio has not been confirmed for urology). In addition, many patients treated with Dysport® experience faster improvement in symptoms treated with BOTOX® (4 days vs. 7 to 10 days for Dysport® and BOTOX®, respectively). Dysport® can be injected deeper and is easier to spread. Therefore, a wider area of the bladder can be treated with Dysport® toxin compared to BOTOX® for a specified period of time. Although some patients have a better response to BOTOX® than Dysport®, the decision to use each of these toxins in different patients varies depending on the patient’s condition. The standard dose of Dysport® was initially based on a conversion fraction of 2.5 to 1 for 2 toxins and was later reduced to 300 units. Most studies on the use of BoNT-A in the treatment of OAB have focused on BOTOX®. Although Dysport® has been shown to be effective in treating patients with NDO,^25^ the use of this toxin in the treatment of bladder diseases should be done off-label. The most common toxin serotype used in the studies was BOTOX®, which is available in the United States and Europe. However, the only serotype available in our country is Dysport.

In order to treat patients with IC/BPS who were resistant to conventional therapy, a multicenter, randomized, double-blind, placebo-controlled trial was done. Hydrodistention plus suburothelial injections of BoNT-A 100 U (Botox group) or an equivalent dose of normal saline (N/S group) were randomly assigned to patients in a 2 : 1 ratio. At week 8 after therapy, the primary endpoint—a reduction in pain—was measured using a visual analog scale (VAS). A total of 60 patients—8 men and 52 women—with an average age of 50.8 years and 13.9 years were enrolled, with 40 in the Botox and 20 in the N/S groups. At week 8, the Botox group experienced a considerably lower level of discomfort than the N/S group (−2.6 2.8 vs. −0.9 2.2, *P* = .021). Except for cystometric bladder capacity, which significantly increased in the Botox group, the remaining measures did not significantly differ across groups. In the Botox group, the overall success rates were 63% (26/40), but in the N/S group, they were 15% (3/20) (*P* = .028). There was no difference in adverse occurrences across the groups.^26^ In a double-blind clinical trial on 21 patients with BPS, only 19 patients completed 12 weeks of follow-up. In the 12th week, a significant reduction in pain was observed in the BOTOX® group compared to the normal saline group. Quality of life was also improved in the BOTOX® group. Worsening of bladder symptoms was observed in the placebo group.^27^ In both studies, the effect of normal saline in the treatment of BPS patients was strong and only a total of 79 patients were included in the 2 studies, which indicates the need for further studies with stronger evidence to confirm the therapeutic effect of BOTOX®.^11^ A significant recurrence of symptoms including pain, urinary frequency, and bladder capacity was observed after 5 months of obobotulinumtoxin injections in the study of Giannantoni et al.^13^

Another study reported a response rate of 38.2% at 6 months and 20.6% at 12 months after intravesical injection of botulinum toxin type A.^28^ Manning et al.^29^ in a double-blind study on 54 women with severe refractory IC, assigned the patients randomly to receive treatment with hydrodistension + injection of sterile saline or hydrodistension + injection of AboBTXA. The ability of nonresponsive patients in either group to get AboBTXA medication made it impossible to conduct additional randomized comparisons after the initial 3 months of measurements. AboBTXA was not associated with a general improvement in the total. The O’Leary-Sant questionnaire consists of problem and symptom index (OLS-PI) scores for patients with chronic refractory IC/BPS; however, some patients showed a significant benefit. A better response to AboBTXA was correlated with the lack of posttreatment UTI.^29^

Our study demonstrated that the majority of cases (93%) had improvement or good response to the treatment after intravesical injection of Dysport®, and only 7% had no response to this therapeutic approach. All of the symptoms according to the BPIC-SS questionnaire were alleviated after treatment. However, we did not find any correlation between pre-treatment symptoms and end outcomes, that is, improvement, good response, or no response after treatment according to the results of Spearman's rank-order correlation coefficient (*P* > .05).

Although obobotulinumtoxin treatment for IC/BPS has not been approved by regulatory authorities, it has been documented in the treatment guidelines of the AUA and Asian Urological Association.^30,31^ In addition, repeated obobotulinumtoxin injections in a 6- to 9-month interval (it should not be shorter than 3 months) for the treatment of lower urinary tract disorders and pelvic floor dysfunction^15^ is recommended. Considering that no serious systemic adverse events have been reported after Botox injection in patients with IC/BPS in the previous documents similar to our findings, besides the beneficial effect of obobotulinumtoxin injection, any potential adverse events of this agent should be considered, and all candidate patients for this approach should be informed about any side effects, and the possibility of CIC should be conveyed.^32^

### Limitations, Drawbacks, and Shortcomings

Recent clinical trials on obobotulinumtoxin injection for the treatment of IC/BPS refractory to conventional therapies have shown promising therapeutic effects including reductions in bladder pain and IC symptoms, but there have been few clinical trials to demonstrate the superiority of obobotulinumtoxin over placebo. In addition, based on our knowledge there is no clinical study to evaluate the effect of Dysport® on IC/BPS, and since our study retrospectively evaluated its effect that was accompanied by promising outcomes, further randomized controlled trials are recommended to investigate its superiority over placebo with considering the need for anesthesia, the occurrence of local complications, risks of urinary retention, and a large PVR volume.

## Conclusion

Our results showed the efficacy and safety of intravesical injections with Abobotulinumtoxin-A (Dysport®) in patients with IC/BPS. Our data show that the treatment is safe and has a high rate of satisfaction, that is, 41% highly satisfied and 52% intermediate satisfaction. Further randomized controlled trials are recommended to investigate its superiority over placebo considering the need for anesthesia, occurrence of local complications, and risks of urinary retention, and a large PVR volume.

## Figures and Tables

**Table 1.** Baseline Characteristics of Eligible Patients

Variables	Mean (SD)	Minimum, Maximum	
Age	57.27 (18.29)	18, 83	
Duration of disease (month)	26.60 (23.46)	6, 120	
Follow-up (month)	9.39 (8.16)	3, 48	
**Gender**	**N (%)**	**UDS results**	N (%)
Male	3 (6.8)	Detrusor overactivity (DO)	3 (6.8)
Female	41 (93.2)	Low capacity	8 (18.2)
**Previous pharmacotherapy**		Low capacity and DO	30 (68.2)
Oral	31 (70.5)	Low capacity, low compliance, and DO	2 (4.5)
Oral + percutaneous nerve evaluation	1 (2.3)	Low capacity and detrusor underactivity (DU)	1 (2.3)
Oral + intravesical infusion	12 (27.3)	**Ending status**	
**Number of Dysport® injection**		No response	3 (6.8)
1	16 (36.4)	Improvement	23 (52.3)
2	3 (6.8)	Good response	18 (40.9)
3	1 (2.3)		

**Table 2.** The Frequency of Symptoms at Baseline and After Treatment

Bladder Pain/Interstitial Cystitis Symptom Score (BPIC-SS)	Never				Rarely				Sometimes				Most of the time						Always			
	Before		After		Before		After		Before		After		Before		After							
1. In the past 7 days when you urinated, how often was it because of pain in your bladder?	1 (2.3)		19 (43.2)		5 (11.4)		22 (50.0)		13 (29.5)		3 (6.8)		22 (50.0)		0 (0.0)				3 (6.8)			
2. In the past 7 days, how often did you still feel the need to urinate just after you urinated?	2 (4.5)		37 (84.1)		20 (45.5)		5 (11.4)		15 (34.1)		2 (4.5)		5 (11.4)		0 (0.0)				2 (4.5)			
3. In the past 7 days, how often did you urinate to avoid pain in your bladder from getting worse?	0 (0.0)		9 (20.5)		4 (9.1)		30 (68.2)		5 (11.4)		5 (11.4)		30 (68.2)		0 (0.0)				5 (11.4)			
4. In the past 7 days, how often did you have a feeling of pressure in your bladder?	2 (4.5)		34 (77.3)		9 (20.5)		8 (18.2)		23 (52.3)		2 (4.5)		8 (18.2)		0 (0.0)				2 (4.5)			
5. In the past 7 days, how often did you have pain in your bladder?	11 (25.0)		35 (79.5)		8 (18.2)		9 (20.5)		10 (22.7)		0 (0.0)		13 (29.5)		0 (0.0)				2 (4.5)			
	**Not at all**				**A little**				**somewhat**				**moderately**						**A great deal **			
6. In the past 7 days, how bothered were you by frequent urination during the daytime?	0 (0.0)		17 (38.6)		3 (6.8)		12 (27.3)		14 (31.8)		15 (34.1)		12 (27.3)		0 (0.0)				15 (34.1)			
7. In the past 7 days how bothered were you by having to get up during the night to urinate?	6 (13.6)		34 (77.3)		11 (25.0)		8 (18.2)		17 (38.6)		2 (4.5)		8 (18.2)		0 (0.0)				2 (4.5)			
n (%)																						
8. The worst bladder pain in the past 7 days	**0 = No bladder pain**		**1**		**2**		**3**		**4**		**5**		**6**		**7**		**8**		**9**		**10=Worst possible bladder pain**	
	Before	After	Before	After	Before	After	Before	After	Before	After	Before	After	Before	After	Before	After	Before	After	Before	After	Before	After
	0 (0.0)	0 (0.0)	0 (0.0)	7 (15.9)	0 (0.0)	21 (47.7)	0 (0.0)	5 (11.4)	0 (0.0)	9 (20.5)	0 (0.0)	2 (4.5)	7 (15.9)	0 (0.0)	2 (47.7)	0 (0.0)	5 (11.4)	0 (0.0)	9 (20.5)	0 (0.0)	2 (4.5)	0 (0.0)

**Table 3.** Correlation Between BPIC-SS Questionnaire Items and Ending Outcomes

Correlations	**Ending Outcome**	
Spearman’s rho	Correlation Coefficient	*P*
1. In the past 7 days when you urinated, how often was it because of pain in your bladder?	0.089	.566
2. In the past 7 days, how often did you still feel the need to urinate just after you urinated?	0.009	.954
3. In the past 7 days, how often did you urinate to avoid pain in your bladder from getting worse?	−0.104	.503
4. In the past 7 days, how often did you have a feeling of pressure in your bladder?	0.014	.930
5. In the past 7 days, how often did you have pain in your bladder?	0.101	.514
6. In the past 7 days, how bothered were you by frequent urination during the daytime?	−0.140	.365
7. In the past 7 days how bothered were you by having to get up during the night to urinate?	−0.088	.570
8. Select the number that best describes your worst bladder pain in the past 7 days	−0.182	.237
Pre-treatment BPIC-SS score	−0.061	.693
